# DOCK8 deficiency and guillain-barre syndrome

**DOI:** 10.1186/1939-4551-8-S1-A167

**Published:** 2015-04-08

**Authors:** Ana Carolina Nápolis, Gesmar Rodrigues Silva Segundo, Marcela Rezende, Laura Carneiro Matoso Nunes Canabrava

**Affiliations:** 1 Universidade Federal De Uberlândia, Brazil

## Background

DOCK8 deficiency has been recently identified as the cause of autosomal recessive hyper IgE syndrome. The presence of autoimmune phenomenas have been described frequently in several primary immunodeficiencies. The aim of this study was to report an interesting case of suspicious DOCK8 deficiency wih characteristic clinical features that presented a classic Guillain-Barre Syndrome.

## Methods

Clinical case report.

## Results

R. I. M., 10 year-old-boy that was referred to us with history of recurrent otitis media, sinusitis, and multiple episodes of pneumonia, palmo-plantar warts, and severe contagious molusco. He also has history of severe atopic dermatitis, asthma and food allergies. On evaluation he was noted to have persistent eosinophilia, lymphopenia, normal IgG levels, absent specific antibody response and decreased CD4 and CD8 T cells. Based on history of severe atopy, sinopulmonary infections, recurrent staphylococcal and viral skin infections DOCK8 deficiency was suspected. Since the suspicious, he started the use of daily cotrimazol and monthly immunoglobulin (500 mg/kg). DOCK8 gene sequencing and duplications/deletions analysis was done and there were found deletions in compound heterozygosis (Alelle 1: deletion of exons 3 to 33; Alelle 2: deletion of exons 16 and 20-24). The diagnosis was confirmed.

At age 11 year, 2 weeks after a viral disease, he started a weakness involving the arms, legs, and truncal muscles, which had a rapid progression (less than 24 h). He performed an investigation with a normal magnetic resonance and the study of nerve conduction showed specific findings consistent with demyelination characteristic for classic Guilain-Barre Syndrome. He received immunoglobulin in a dose of 2g/kg and evaluated with resolution of the neurologic symptoms. Currently, he keeps receiving monthly immunoglobulin and prophylactic cotrimazol daily and showed no new serious infections. It was found a compatible bone marrow donor and he is being prepared to transplantation.

## Conclusions

DOCK8 deficiency is a primary immunodeficiency that has to be suspected facing a case with characteristic clinical features. It can evaluate with autoimmune complications like others primary immunodeficiencies.

## Consent

Written informed consent was obtained from the patient for publication of this abstract and any accompanying images. A copy of the written consent is available for review by the Editor of this journal.

